# Parameters of the Immune System and Vitamin D Levels in Old Individuals

**DOI:** 10.3389/fimmu.2018.01122

**Published:** 2018-05-24

**Authors:** Amanda Soares Alves, Mayari Eika Ishimura, Yeda Aparecida de Oliveira Duarte, Valquiria Bueno

**Affiliations:** ^1^Division of Immunology, DMIP Microbiology, Immunology, and Parasitology, Federal University of São Paulo, São Paulo, Brazil; ^2^Division of Epidemiology, Faculdade de Saúde Pública, Universidade de São Paulo, São Paulo, Brazil

**Keywords:** longevity, immunity, vitamin D, myeloid-derived suppressor cells, T cells

## Abstract

**Aim:**

The increased number of individuals older than 80 years, centenarians, and supercentenarians is not a synonym for healthy aging, since severe infections, hospitalization, and disability are frequently observed. In this context, a possible strategy is to preserve the main characteristics/functions of the immune system with the aim to cause less damage to the organism during the aging process. Vitamin D acts on bone marrow, brain, breast, malignant cells, and immune system and has been recommended as a supplement. We aimed to evaluate whether immune parameters and vitamin D serum levels are correlated.

**Methods:**

We evaluated some features of the immune system using the peripheral blood of individuals older than 80 years (*n* = 12) compared to young subjects (*n* = 10). In addition, we correlated these findings with vitamin D serum levels.

**Results:**

Old individuals presented metabolic parameters of healthy aging and maintained preserved some features of immunity such as CD4/CD8 ratio, and low production of pro-inflammatory cytokines after stimulus. On the other hand, we observed increase in the frequency of myeloid-derived suppressor cells, reduction in circulating leukocytes, in the percentage of total CD8+, and in CD8+ Naïve T cells, in addition to increase in the percentage of CD8+ effector memory re-expressing CD45RA (EMRA) T cells. We found seropositivity for CMV in 97.7%, which was correlated with the decrease of CD8+ Naïve T cells and increase in CD8+ EMRA T cells. Vitamin D levels were insufficient in 50% of old individuals and correlated positively with total CD8+ T cells and negatively with CD8+ EMRA T cells.

**Conclusion:**

In the studied population, longevity was correlated to maintenance of some immune parameters. Considering the limitations of the study as size of the sample and lack of functional assays, it was found that vitamin D in old individuals was correlated to some features of the immune system, mainly in the CD8 compartment.

## Introduction

In several developed and developing countries, human longevity has been achieved (1–3) but insufficient function of the immune system in the old population leads to severe infections, frequent hospitalizations, and immunization reduced after vaccination (4–6).

Therefore, to reach the longevity with good quality of life, a possible strategy is to preserve the main characteristics/functions of the immune system with the aim to cause less damage to the organism during the aging process. Aging has been associated with lower generation of progenitors from pluripotent hematopoietic stem cells (HSC) located in the bone marrow. In addition, there occurs a myeloid-biased differentiation of HSC due to selection pressures from cell-intrinsic and extrinsic mechanisms (7–10). These factors lead to a decrease in the number of circulating leukocytes and increased frequency in the myeloid lineage. The increase in the frequency of myeloid cells may be the reason for the recently described increase in myeloid-derived suppressor cells (MDSC) in aging individuals. MDSC membrane markers are CD11b^+^CD33^+^HLA^−^DR^−/low^, which can be subtyped in monocytic MDSC (CD14^+^) or granulocytic (CD15^+^) MDSC.

The suppressive actions of MDSC are mainly based on the production of arginase-1, reactive oxygen species, nitric oxide (NO), IL-10, and TGF-β1. These cells exert several effects on lymphocytes such as impairment in the antigen presentation and recognition by T cells, deficient B and T cells activation, and accumulation of regulatory T cells (11, 12).

Changes in hematopoiesis have also been related to the decreased percentage of CD4^+^ and CD8^+^ T cells in the circulating blood. In addition, the inverted CD4/CD8 ratio was reported by Swedish OCTO and NONA immune longitudinal studies as a blood marker predictive for a high rate of mortality in 2, 4, and 6 years (immune risk profile) (13).

Another aspect of aging is the thymic involution which has been linked to less diversity in the T-cell receptor and decreased frequency of Naive T cells (14, 15), while the peripheral homeostatic proliferation compensates for the T-cell loss (16, 17).

The phenotype of T cells has been used to characterize the immune system status and Hamann et al. (18, 19) proposed that in humans, the CD8^+^ T cell compartment presents four different phenotypes based on membrane markers and cellular function. The phenotype CD45RA^+^CD27^+^ represents undifferentiated Naive cells and CD45RA^−^CD27^+^ lymphocytes are antigen-experienced T cells (central memory) with increased frequency of lymphotoxicity precursors (CTLp). CD45RA^+^CD27^−^ cells present features of antigen-stimulated cells with cytolytic potential, production of IFN-γ and tumor necrosis factor-alpha (TNF-α), high amounts of perforin and granzyme B, Fas ligand mRNA expressed in abundancy, and cells exerting potent cytotoxic activity without previous *in vitro* stimulation [effector memory re-expressing CD45RA (EMRA)]. CD45RA^−^CD27^−^ T cells are observed in low frequency and express perforin and granzyme B (effector memory). The correlation between aging and increased frequency of CD8^+^CD45RA^+^CD27^−^ has been reported. The same phenotypes were identified in different stages of CD4^+^ T cells mainly during stimulation by viral infections (CMV, EBV, HSV, and VZV) (20, 21).

In order to preserve the functional characteristics of the immune system that could in turn prevent and/or delay age-related diseases, health professionals have proposed physical activity, control of diet, supplements, and probiotics (22–24).

The main actions of vitamin D in bone tissue and the latest reports of its effects on bone marrow, brain, colon, breast, malignant cells, and immune system (25) raised the interest of researchers to investigate the role played by vitamin D in the immunity of old individuals.

Considering that low levels of vitamin D are common in older individuals, some health professionals have recommended vitamin D supplementation to the aging population in general and especially for aged-care residents and critically ill patients (26–28). However, the benefits arising on the immunity with vitamin D supplementation are not consistent in the literature. Upper respiratory infections (URI) in non-hospitalized middle-aged and older individuals with vitamin D supplementation have been associated with a lower incidence of infection (29), discrete decrease of infections events (30), or no alteration in severity and duration of infection (31, 32). In addition, residents of sheltered accommodation supplemented with vitamin D showed increase in URI and duration of symptoms and no changes to the risk or duration of lower respiratory infections (33). However, in patients with antibody deficiency or increased susceptibility to respiratory tract infections (RTI), supplementation with vitamin D was beneficial and associated with fewer episodes of RTI and increased time for first infection compared to placebo group (34).

As the extension of life expectancy is a reality, it is a challenge to understand how the aging population deals with the remodeling of the immune system and if interventions as vitamin D could provide extra years of life with good health. In this study, our goal was to investigate changes that occur in some parameters of the immune system in individuals reaching longevity (80–100 years) and the possible correlation with vitamin D levels.

## Materials and Methods

The present study is part of a larger epidemiologic survey called the health, well-being, and aging study (SABE), which was coordinated by the Pan-American Health Organization, Washington, and in Brazil by the School of Public Health of the University of São Paulo. From 2000 to 2001, SABE evaluated a sample of 2,143 non-institutionalized individuals, representing 836,204 aging people (60 years and older) living in the municipality of São Paulo, who were selected through multi-stage sampling. In 2006, the School of Public Health continued the survey in São Paulo and transformed it into a multi-cohort study with 1,115 individuals from the previous study who agreed to participate in the follow-up. Since then, the survey has been repeated every 5 years. In this study, the inclusion/exclusion criteria were applied as cited above, except that we used blood only from individuals older than 80 years (male, female) and they were enrolled as their biological samples were received. Young individuals (20–30 years, male and female) were master and Ph.D. students from UNIFESP.

The blood samples were collected with 12 h of fasting (6:00 a.m.–9:00 a.m.) during the summer months (December, January, and February) in Brazil.

The Ethics Committee of the Federal University of São Paulo—UNIFESP approved all procedures (Protocol number 10904).

Peripheral blood mononuclear cells (PBMCs) were isolated using Ficoll–Hypaque density gradient (Amersham Biosciences, Uppsala, Sweden) and centrifugation. Viable cells were counted, adjusted for 2 × 10^6^/100 μL in 80% fetal bovine serum and 20% dimethylsulfoxide (Sigma, St. Louis, MO, USA), and frozen stored (−80°C) until the phenotyping and cell culture.

### Cell Culture

Cells were diluted in RPMI 10, counted, and adjusted for 1 × 10^6^/100 mL. The assessment of cell proliferation was based on a substance (CFSE) that once in the cell cytoplasm halves its content to each cell division. Cells were incubated with 5(6)-carboxyfluorescein acetate (CFSE, CFDA Vybrant IF Cell Tracer Kit Invitrogen) for 10 min. The cells were washed, counted, and adjusted for plating (2 × 10^5^/100 μL of RPMI10 per well). Culture conditions were phytohemagglutinin (PHA^+^, 5 µg/mL, Sigma) or absence of stimulus (PHA) for 72 h in 5% CO_2_, humidity controlled and 37°C. After 3 days of culture, the cell suspension was collected, centrifuged, and the proliferation (CFSE) was measured in flow cytometer (35). The cell culture supernatant was frozen (−80°C) for the measurement of cytokines (ELISA).

### Cell Phenotype

The cells were stained with monoclonal antibodies to T-cell phenotype CD3 APC, CD4 PerCP Cy 5.5, CD8 APC Cy7, CD27 FITC, CD45RA PE (eBioscience, CA, USA). The cells were also stained with monoclonal antibodies to MDSC phenotype CD3 APC, CD19 APC, CD56 APC, HLA-DR APC e-fluor 780, CD33 PerCP Cy5.5, CD11b PE, CD14 PE Cy7, CD15 FITC (eBioscience, CA, USA). After 30 min of incubation with monoclonal antibodies, in the dark and at 4°C, the cells were washed with PBS and centrifuged. Living cells (based on forward and side scatter) were acquired in the FACS Canto II using the DIVA software (Becton Dickinson, USA). Further analyses of FACS data were performed using the 9.3 FLOWJO software (Tree Star, USA).

T lymphocytes were characterized as described previously (36).

Naïve: CD3^+^CD4^+^CD45RA^+^CD27^+^ or CD3^+^CD8^+^CD45RA^+^CD27^+^ (Naïve).
Central memory: CD3^+^CD4^+^CD45RA^−^CD27^+^ or CD3^+^CD8^+^CD45RA^−^CD27^+^ (CM).
Effector memory: CD3^+^CD4^+^CD45RA^−^CD27^−^ or CD3^+^CD8^+^CD45RA^−^CD27^−^ (EM).
Effector memory re-expressing CD45RA: CD3^+^CD4^+^CD45RA^+^CD27^−^ or CD3^+^CD8^+^CD45RA^+^CD27^−^ (EMRA).

Myeloid-derived suppressor cells were characterized as:

CD3^−^CD19^−^CD56^−^HLA^−^DR^−/low^CD33^+^CD11b^+^CD15^+^ granulocytic or
CD3^−^CD19^−^CD56^−^HLA^−^DR^−/low^CD33^+^CD11b^+^CD14^+^ monocytic.

### ELISA

The frozen culture supernatants were thawed and cytokines [IL-1, IL-2 α, interleukin-6 (IL-6), IFN-γ, and TNF-α] were evaluated by ELISA assay according to the manufacturer’s instructions (DuoSet ELISA Development Systems R&D). ELISA reading PerkinElmer—EnSpire, quantified the samples.

### Metabolic Data

Obtained from the databank of SABE study.

### Cytomegalovirus IgM and IgG

Serum was previously isolated by centrifugation and frozen stored (−80°C). IgG and IgM levels were measured in serum by electrochemiluminescence immunoassay according to the manufacturer’s instructions (cobas^®^ http://e-labdoc.roche.com REF 04784618 190).

### Measurement of Vitamin D

Serum of studied individuals was previously isolated by centrifugation and frozen stored (−80°C) until the measurement of vitamin D. 25-Hydroxyvitamin D value was obtained in accordance with the manufacturer’s instructions (cobas^®^ http://e-labdoc.roche.com 05894913 190 V7).

### Statistics

After testing the variables for normality (Shapiro–Wilk) it was used the unpaired Student’s *t*-test (Vitamin D) or Mann–Whitney (parameters of the immune system) for comparisons between young and old groups. The correlation between parameters of the immune system and serum levels of IgG (CMV) or vitamin D was performed by Spearman test. Values of *p* < 0.05 were considered statistically significant. All statistical analyses were performed with the aid of the Graph Pad PRISM software (Graph pad, La Jolla, CA, USA).

## Results

Young (*n* = 10, 5 male and 5 female, 20–30 years old) and old (*n* = 12, 6 male and 6 female, 80–100 years old) individuals were evaluated. Table 1 shows some metabolic parameters that highlight the health status of the old population evaluated. In agreement with Helmersson-Karlqvist et al. (37), our old population may be considered healthy for most of the parameters evaluated in spite of great variability observed. Only one female (100 years) presented glucose (162 mg/dL) higher than the reference values established by Helmersson-Karlqvist et al. (37).

**Table 1:** Metabolic parameters in old individuals (80–100 years) and reference values of 80-year-old individuals (37).

Metabolic parameters								
Age	Gender	Cholesterol total mg/dL	HDL mg/dL	Triglycerides mg/dL	Glucose mg/dL	Urea mg/dL	Creatinine mg/dL	Albumin mg/dL
100	F	179	38	183	162	35	0.91	3.1
90	F	224	53	178	80	33	1.09	3.4
90	F	188	73	125	86	42	0.67	3.9
93	M	181	43	47	80	57	0.98	3.9
93	F	186	51	121	93	28	0.93	3.6
88	M	275	68	114	104	36	1.01	4
94	M	177	39	86	71	47	1.26	3.4
90	M	163	43	72	82	30	1.1	3.7
90	M	181	38	135	95	43	1.22	3.9
94	M	132	33	80	90	61	1.97	3.9
91	F	207	63	106	81	41	0.73	4.3
90	F	236	58	109	90	29	0.94	4

Mean ± SD		194.1±37.1	50 ± 13.1	113 ± 40.2	92.8 ± 2	40.2 ± 10.7	1.1 ± 0.3	3.8 ± 0.3
Range		132–275	33–73	47–183	71–162	28–61	0.67–1.97	3.1–4.3
Reference values (37)		108.3–313.2	27.1–104.4	43.8–287.8	126	19.8–89.5	0.52–1.94	3.2–4.7

**Figure 1:**
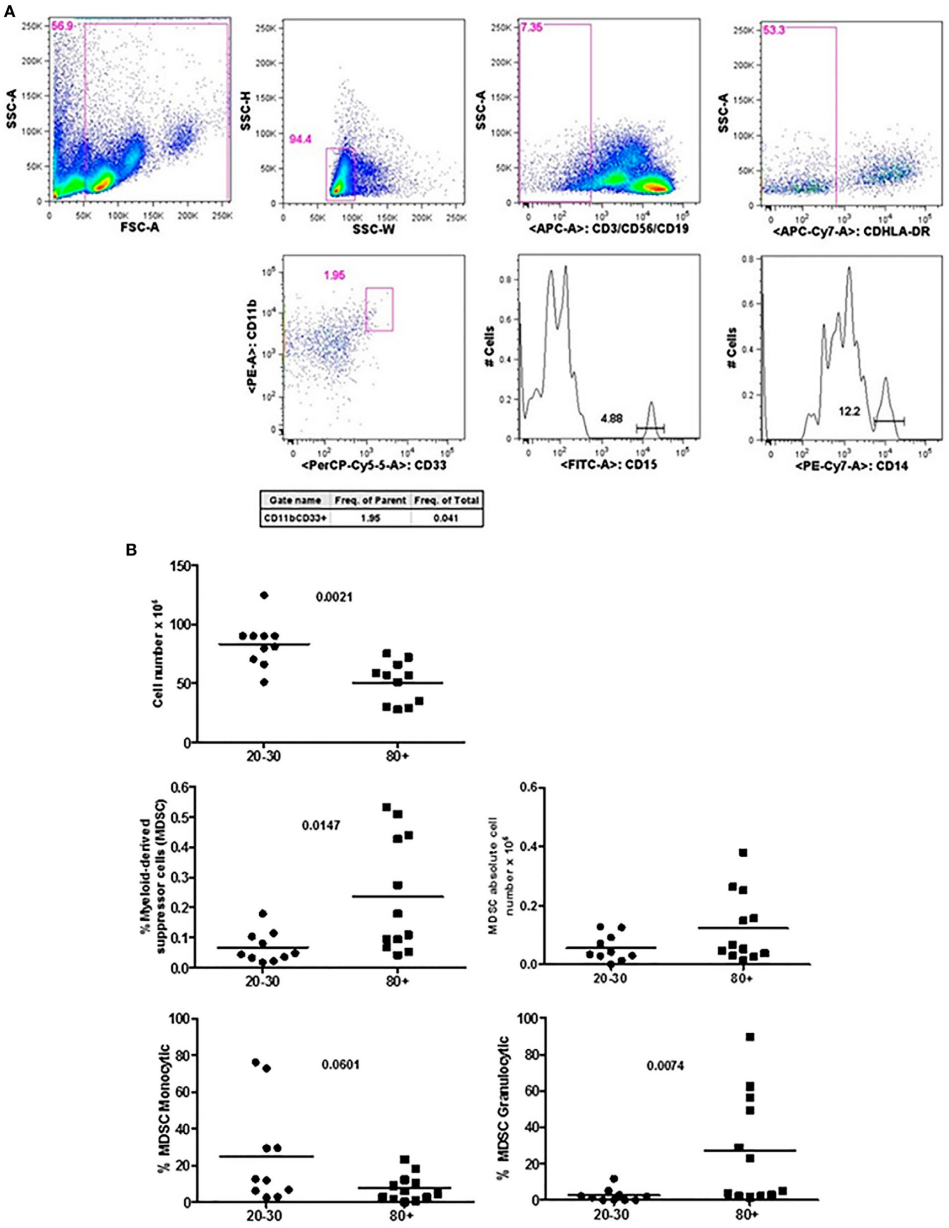
Representative flow cytometry plots showing gate strategy for myeloid-derived suppressor cells (MDSC) frequency. Gate in live cells (FSC-A × SSC-A), doublets exclusion (SSC-H × SSC-W), gate in lineage negative cells (CD3^−^CD56^−^CD19^−^), gate in HLA-DR^low/neg^ cells, gate in CD33^+^CD11b^+^ cells. Gate in granulocytic (CD15^+^) and monocytic (CD14^+^) cells **(A)**. Number of circulating leukocytes, MDSC (% MDSC) frequency, absolute number of MDSC, and frequency of monocytic and granulocytic MDSC in individuals of 20–30 years and 80+ years old individuals **(B)**.

**Figure 2:**
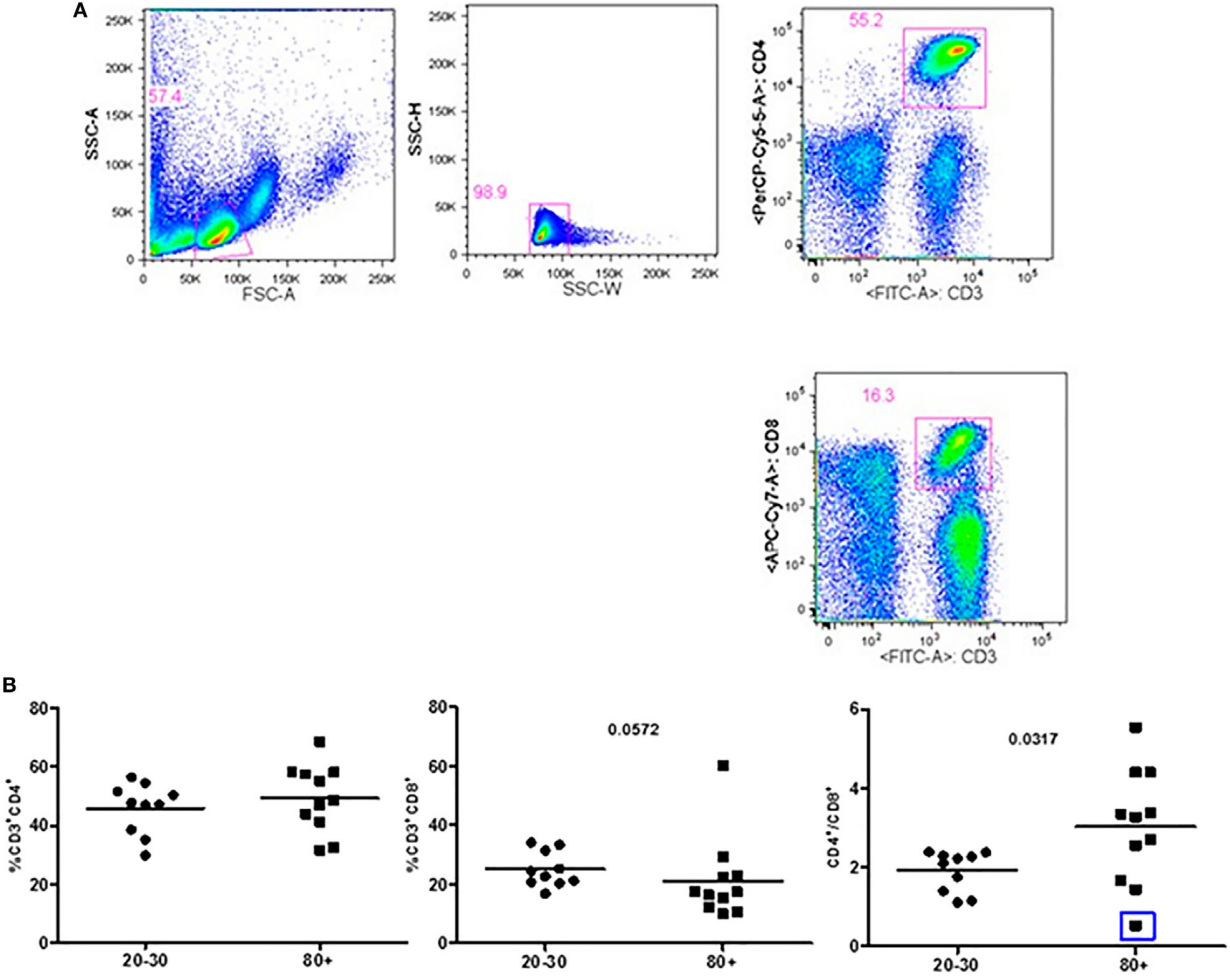
Representative flow cytometry plots showing gate strategy for the frequency of CD4^+^ and CD8^+^ T cells. Gate in live lymphocytes (FSC-A × SSC-A), doublets exclusion (SSC-H × SSC-W), gate in CD3^+^CD4^+^ T cells, gate in CD3^+^CD8^+^ T cells **(A)**. Frequency of T lymphocytes CD3^+^CD4^+^, CD3^+^CD8^+^, and CD4/CD8 ratio in 20–30 and 80^+^ years old individuals. The CD4/CD8 ratio lower than 1 (0.542; blue square) **(B)**.

Figure 1 shows that old individuals (80–100 years) presented a significantly high percentage of MDSC (%MDSC). However, as this group had a significantly lower number of leukocytes, the absolute cell number of MDSC (leukocytes cell number × percentage of MDSC) was not different compared to young individuals (20–30 years). There was significant predominance of granulocytic MDSC in individuals older than 80 years, while the subtype monocytic was statistically higher in young individuals. The percentage of T CD4^+^ lymphocytes was similar in both groups while there was a trend toward lower percentage of CD8^+^ T cells in old individuals (*p* = 0.0572). The CD4/CD8 ratio was significantly higher in old individuals except in a woman (96 years) with the CD4/CD8 ratio inverted (0.542) (Figure 2).

**Figure 3:**
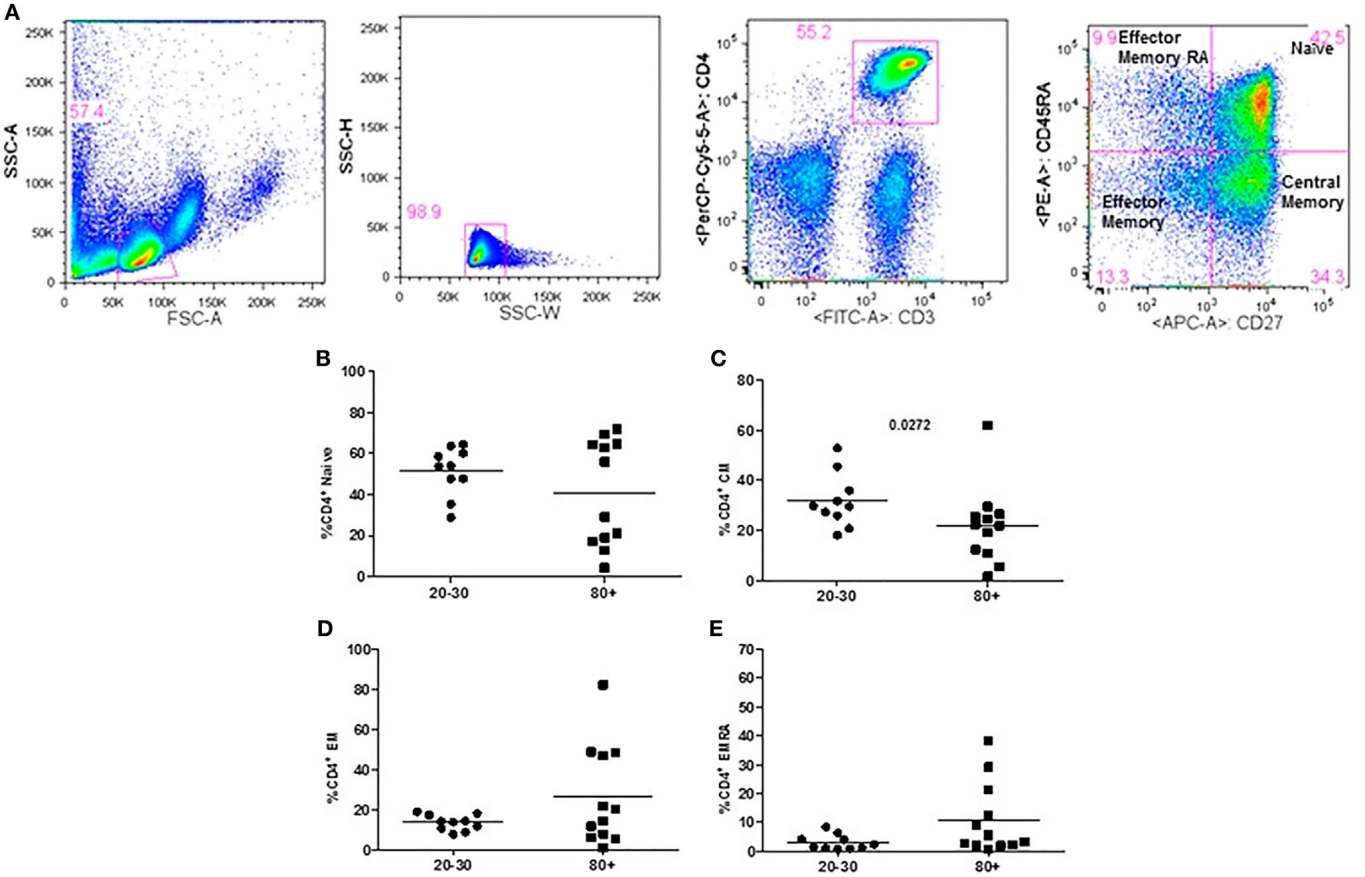
Representative flow cytometry plots showing gate strategy for the frequency of CD4^+^ T cells phenotype. Gate in live lymphocytes (FSC-A × SSC-A), doublets exclusion (SSC-H × SSC-W), gate in CD3^+^CD4^+^ T cells, gate in CD45RA^+^CD27^+^ (Naïve) T cells, gate in CD45RA^−^CD27^+^ (central memory) T cells, gate in CD45RA^−^CD27^−^ (effector memory), gate in CD45RA^+^CD27^−^ (effector memory re-expressing CD45RA) T cells **(A)**. Percentage (%) of CD4^+^ T cells phenotypes: Naïve **(B)**, central memory **(C)**, effector memory **(D)**, effector memory RA re-expressing CD45RA **(E)** in 20–30 and 80+ years old individuals.

**Figure 4:**
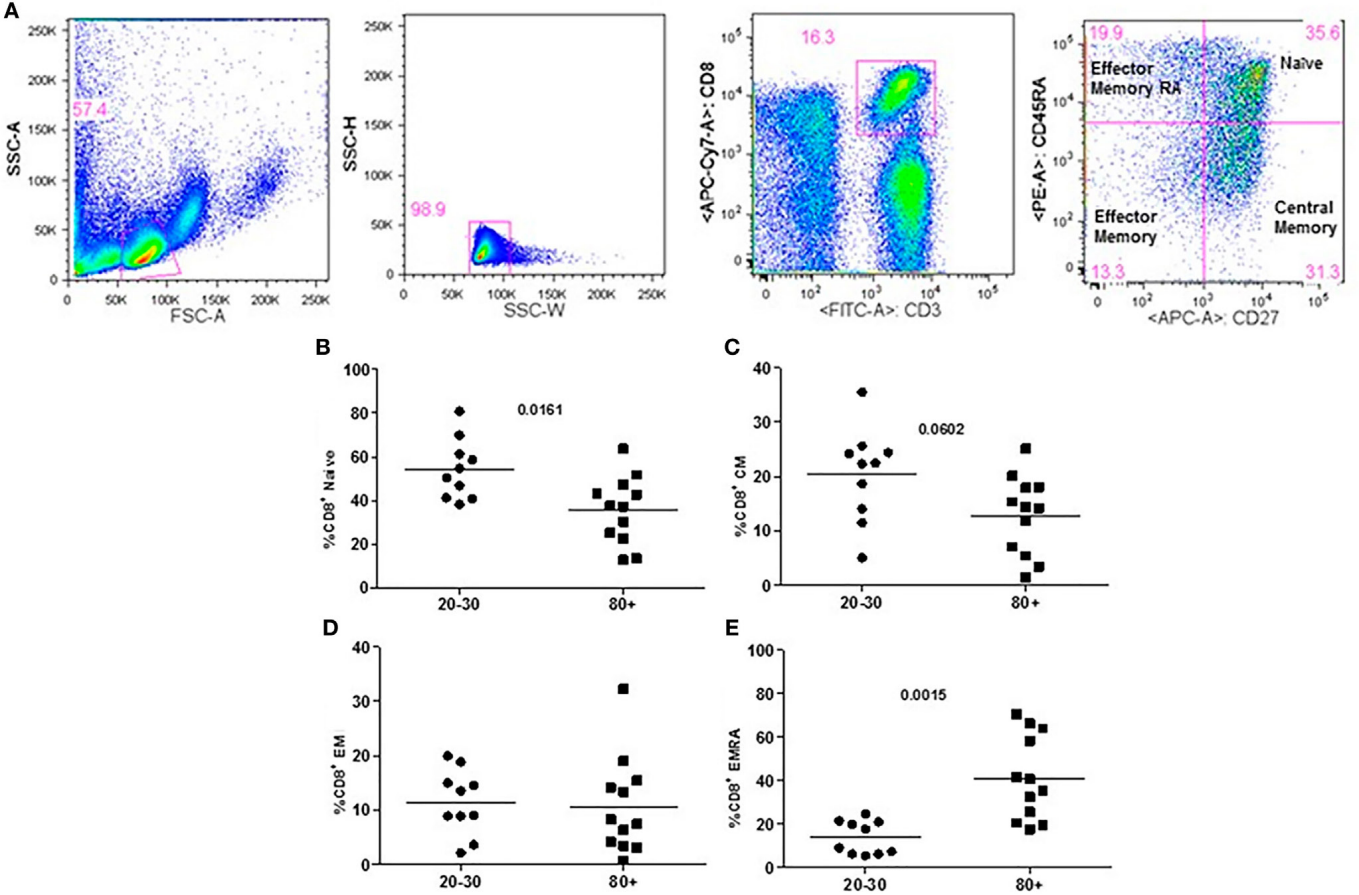
Representative flow cytometry plots showing gate strategy for the frequency of CD8^+^ T cells phenotype. Gate in live lymphocytes (FSC-A × SSC-A), doublets exclusion (SSC-H × SSC-W), gate in CD3^+^CD8^+^ T cells, gate in CD45RA^+^CD27^+^ (Naïve) T cells, gate in CD45RA^−^CD27^+^ (central memory) T cells, gate in CD45RA^−^CD27^−^ (effector memory), gate in CD45RA^+^CD27^−^ (effector memory re-expressing CD45RA) T cells **(A)**. Percentage (%) of CD8^+^ T cells phenotypes: Naïve **(B)**, central memory **(C)**, effector memory **(D)**, effector memory RA re-expressing CD45RA **(E)** in 20–30 and 80+ years old individuals.

**Figure 5:**
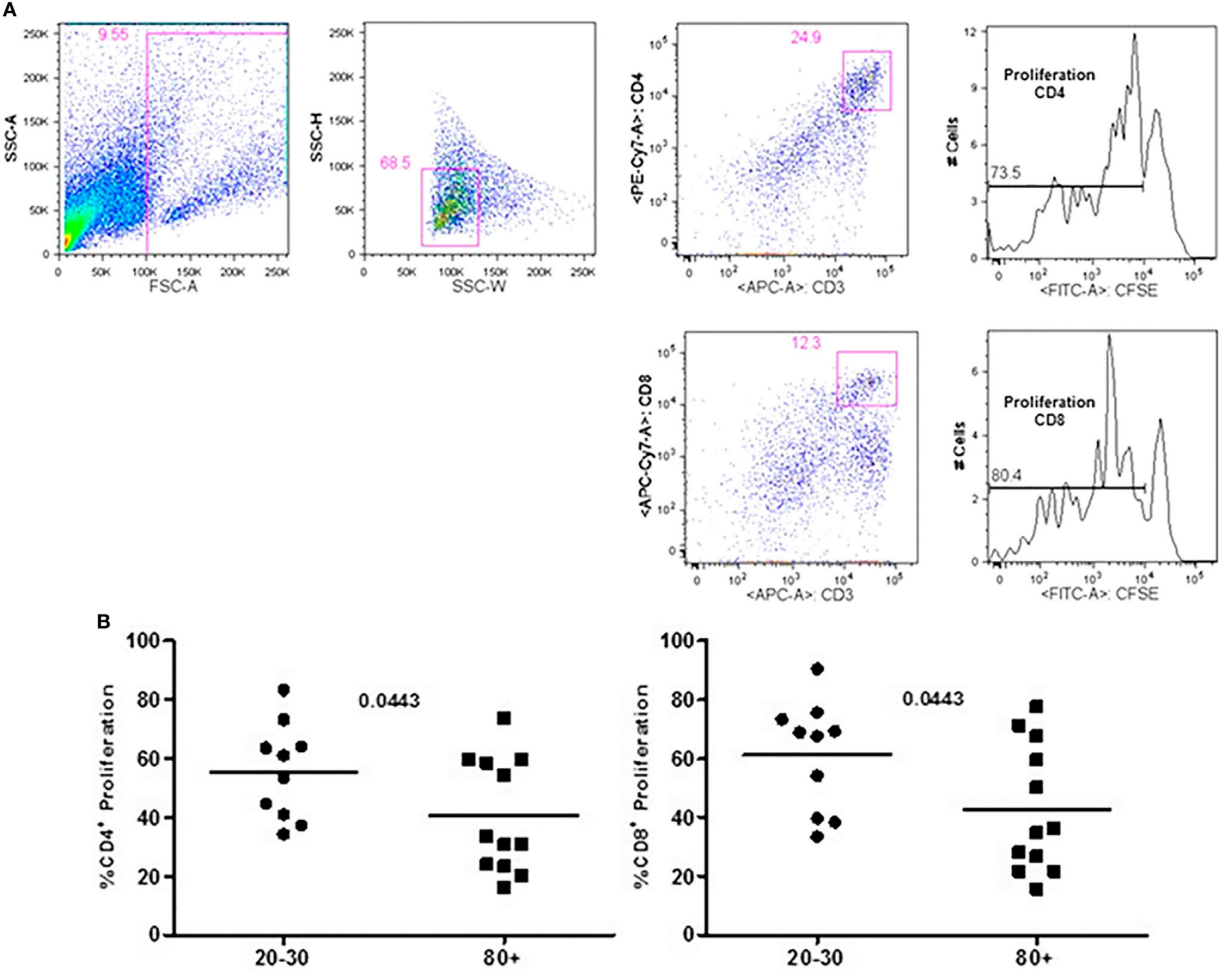
Representative flow cytometry plots showing gate strategy for the frequency of proliferation in CD4^+^ and CD8^+^ T cells after stimulus with phytohemagglutinin (PHA) in culture. Gate in live cells (FSC-A × SSC-A), doublets exclusion (SSC-H × SSC-W), gate in CD3^+^CD4^+^ T cells, gate in the decrease of CFSE, gate in CD3^+^CD8^+^ T cells, gate in the decrease of CFSE **(A)**. Percentage of CD3^+^CD4^+^ and CD3^+^CD8^+^ T cells proliferation after stimulus with PHA according to age: 20–30 and 80+ years old **(B)**.

**Figure 6:**
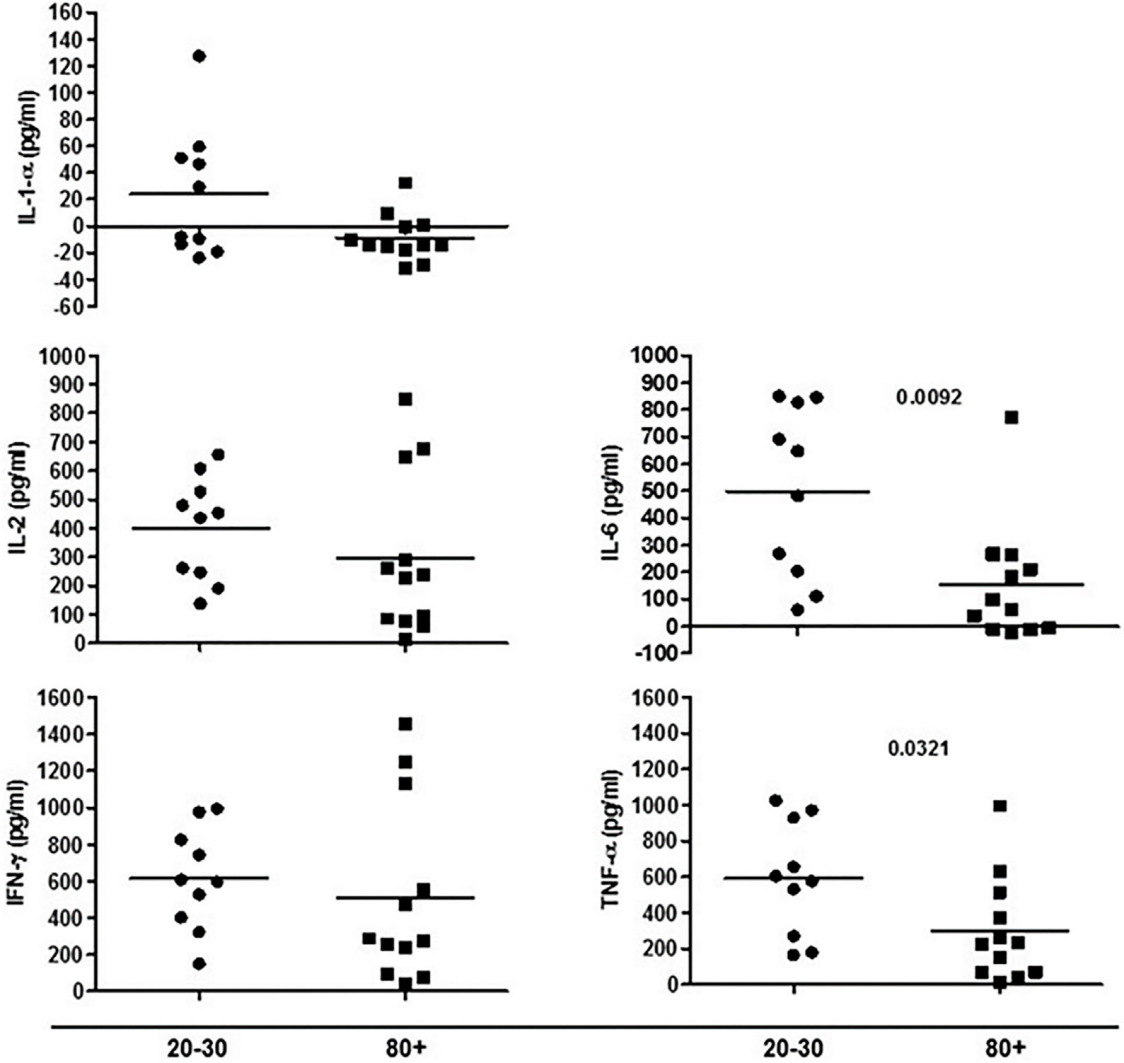
Cytokines produced by cells in culture under phytohemagglutinin stimulus according to age: 20–30 and 80+ years old.

The evaluation of T cells phenotypes showed a significantly lower percentage of CD4^+^ central memory in elderly individuals (Figure 3). In the CD8 compartment, Naive cells were statistically less expressed in old individuals and CD8^+^ EMRA T cells presented significantly higher expression in old individuals (Figure 4). Considering the functions of T cells after stimulation in culture with PHA, the percentage of proliferation was lower in old individuals both in the compartment CD4^+^ and CD8^+^ (Figure 5). The production of cytokines was reduced in old individuals with statistical difference for IL-6 and TNF-α (Figure 6). As cytomegalovirus infection/latency has been related to immunosenescence, we evaluated IgM and IgG against CMV in serum of old (80–100 years) individuals (Table 2) and the possible correlation with immune parameters (Table 3).

**Table 2:** Serum levels of IgM and IgG to cytomegalovirus in old (80–100 years) individuals.

Age	Gender	IgM	IgG
100	F	0.159	>500
90	F	0.296	240.60
90	F	0.164	>500
93	M	0.237	316.30
93	F	0.160	31.36
88	M	0.158	<0.25
94	M	0.436	153.90
90	M	1.240	55.85
90	M	0.189	>500
94	M	0.157	180.60
91	F	0.488	>500
90	F	0.428	87.39

*IgM cutoff index negative: <0.7; indeterminate: 0.7–0.99; positive: ≥1.0*.

*IgG negative <0.5 U/mL; indeterminate: 0.5–0.99 U/mL; positive: ≥1.0 U/mL*.

**Table 3:** IgG levels against cytomegalovirus and correlation with parameters of the immune system in old (80–100 years) individuals.

Parameters	*r* Spearman	*p*
Cell number × 10^5^	−0.164	0.6054
% Myeloid-derived suppressor cells (MDSC) (CD33^+^CD11b^+^)	0.071	0.8267
MDSC absolute cell number × 10^5^	0.046	0.883
% CD15 MDSC	−0.267	0.397
% CD14 MDSC	−0.377	0.225
% CD3^+^CD4^+^	−0.209	0.532
% CD3^+^CD8^+^	0.107	0.753
CD4/CD8	−0.191	0.566
% Proliferation CD4^+^	0.259	0.410
% Proliferation CD8^+^	0.085	0.792
% CD4^+^ Naïve	−0.135	0.673
% CD4^+^ central memory	−0.559	0.06
% CD4^+^ effector memory	0.295	0.347
% CD4^+^ effector memory RA	0.499	0.100
% CD8^+^ Naïve	−0.648	0.027
% CD8^+^ central memory	−0.420	0.174
% CD4^+^ effector memory	0.085	0.792
% CD8^+^ effector memory RA	0.573	0.055
IL-1α	−0.346	0.276
IL-2	0.025	0.941
Interleukin-6	0.143	0.652
IFN-γ	0.110	0.731
Tumor necrosis factor-alpha	0.075	0.818
Vitamin D	0.302	0.336

Table 2 shows that in 12-old individuals evaluated, only one male (88 years old) can be considered negative for cytomegalovirus as the IgM level was <0.7 (0.158) and IgG level was <0.5 (<0.25). In addition, only one male (90 years old) could be considered as recently infected by CMV as the IgM level was >1.0 (1.240) and the IgG level was relatively low (55.85) in comparison to other old individuals studied. Levels of IgG >500 U/mL were observed in four individuals (3 females and 1 male).

The levels of IgG against CMV were negatively correlated (*p* = 0.027) with the percentage of Naïve CD8^+^T cells. There was a trend of negative correlation (*p* = 0.06) between the percentage of CD4^+^ central memory T cells and IgG levels (CMV). A trend of positive correlation (*p* = 0.055) between the percentage of CD8^+^ EMRA T cells and IgG levels (CMV) was observed (Table 3).

**Figure 7:**
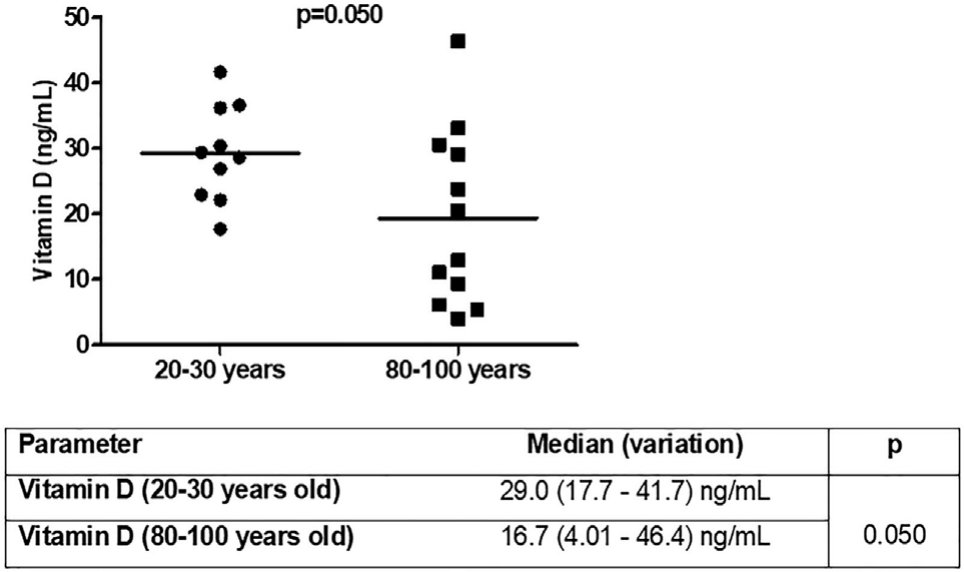
Vitamin D [25(OH)D3] levels in young (*n* = 10; 20–30 years) and old (*n* = 12; 80–100 years) individuals.

The next question was whether total vitamin D levels were different in the studied groups (Figure 7). Vitamin D levels were lower in old individuals (*p* = 0.050). In 50% (*n* = 6) of aged individuals vitamin D levels were <20 ng/mL (deficiency) and in 90% (*n* = 9) of young individuals vitamin D levels were greater than 20 ng/mL. Insufficiency (21–29 ng/mL) was present in 25% (*n* = 3) of old individuals and 50% (*n* = 5) of young individuals. Sufficient levels (30 ng/mL or more) were observed in 25% (*n* = 3) of aged and 40% (*n* = 4) of young individuals.

It was analyzed whether total vitamin D was correlated with the immunological parameters evaluated previously. We observed correlation of vitamin D levels only for the CD8 compartment (Table 4). The percentage of total CD8 T cells was positively correlated (*p* = 0.006) with vitamin D levels in old individuals, whereas there was a trend toward positive correlation (*p* = 0.074) in young individuals. CD8^+^ effector memory T cells were positively correlated with vitamin D levels in young individuals and CD8^+^ EMRA T cells were negatively correlated (*p* = 0.05) with vitamin D levels in old individuals.

**Table 4:** Vitamin D [25(OH)D3] levels and correlation with parameters of the immune system in young (20–30 years) and old (80–100 years) individuals.

Parameters	*r* Spearman 20–30 years	*p*	*r* Spearman 80–100 years	*p*
Cell number × 10^5^	0.012	0.973	−0.182	0.571
% Myeloid-derived suppressor cells (MDSC) (CD33^+^CD11b^+^)	−0.527	0.117	0.370	0.235
MDSC absolute cell number × 10^5^	−0.563	0.089	0.181	0.571
% CD15 MDSC	0.281	0.431	0.167	0.602
% CD14 MDSC	−0.176	0.626	0.168	0.602
% CD3^+^CD4^+^	0.442	0.200	0.190	0.574
% CD3^+^CD8^+^	0.588	0.074	0.764	0.006
CD4/CD8	−0.090	0.803	−0.547	0.082
% Proliferation CD4^+^	0.345	0.328	−0.238	0.457
% Proliferation CD8^+^	0.236	0.511	−0.126	0.697
% CD4^+^ Naïve CD3^+^CD4^+^CD45RA^+^CD27^+^	−0.109	0.764	−0.027	0.931
% CD4^+^ central memory CD3^+^CD4^+^CD45RA^−^CD27^+^	−0.079	0.829	0.287	0.366
% CD4^+^ effector memory CD3^+^CD4^+^CD45RA^−^CD27^−^	−0.042	0.907	0.028	0.931
% CD4^+^ effector memory RA CD3^+^CD4^+^CD45RA^+^CD27^−^	0.150	0.701	−0.192	0.548
% CD8^+^ Naïve CD3^+^CD8^+^CD45RA^+^CD27^+^	−0.297	0.405	0.378	0.269
% CD8^+^ central memory CD3^+^CD8^+^CD45RA^−^CD27^+^	0.236	0.511	0.462	0.131
% CD8^+^ effector memory CD3^+^CD8^+^CD45RA^−^CD27^−^	0.697	0.025	0.260	0.467
% CD8^+^ effector memory RA CD3^+^CD8^+^CD45RA^+^CD27^−^	0.188	0.603	−0.552	0.050
IL-1α (pg/mL)	−0.472	0.169	0.505	0.094
IL-2 (pg/mL)	0.030	0.934	−0.266	0.404
Interleukin-6 (pg/mL)	−0.151	0.676	−0.246	0.442
IFN-γ (pg/mL)	−0.491	0.150	−0.014	0.966
Tumor necrosis factor-alpha (pg/mL)	0.212	0.556	−0.133	0.681

## Discussion

Morbidities associated with aging contribute to organ failure that leads to a pathway of poor quality of life and/or death. In addition, the impairment in the protective functions of the immune system promoting infections and tumors, and the increase of inflammatory factors causing tissue damage and contributing for age-related diseases have also been reported in old individuals.

In our study, it was observed that old individuals presented metabolic parameters consistent with healthy aging except for a female (100 years) with blood glucose higher than 126 mg/dL. Our data are in agreement with the report of Helmersson-Karlqvist et al. (37) who have developed reference intervals of metabolic parameters adjusted for very old (80 years) individuals.

Despite our studied population could be considered healthy, we observed characteristics reported as immune senescence.

Myeloid-derived suppressor cells were only recently described as potential markers of the aging process, since these cells were initially associated with tumor development (12). We observed in old individuals that MDSC were present in high percentage with predominance of the granulocytic subtype. It has been reported that the accumulation of MDSC with aging may contribute to some of the immune disorders and pathologies observed in older adults (12). In aging individuals, Verschoor et al. observed a significant increase in the frequency of MDSC compared to young adults in addition to the higher number of MDSC in older individuals with frailty and previous history of cancer (11). In addition to the increased percentage of MDSC, our old population presented decreased number of circulating leukocytes, reduced percentage of total CD8^+^ and CD8^+^ Naïve T cells, and increase in the percentage of terminally differentiated CD8^+^ EMRA T cells. Significant changes in the phenotype and function occurred mainly in CD8^+^ T cells in these individuals and are in agreement with the literature (38–43). In addition, it has been shown that the homeostatic proliferation is less effective for CD8 than for CD4 Naïve T cells (44).

In old individuals, it has been shown that many aspects of immunosenescence are related to the seropositivity for cytomegalovirus (CMV). However, it seems that the effects of CMV in the immune system of healthy old individuals are dependent on the increased latency of the virus (45, 46). In our study, 11 out of 12 old individuals were seropositive for CMV and four individuals presented IgG levels >500 U/mL. The seropositivity to CMV was correlated with the decrease of Naïve CD8^+^ T cells and with a trend toward decrease in CD4^+^ central memory T cells and increase in CD8^+^ EMRA T cells. The impact of CMV infection/latency in immunity can exacerbate the features of immunosenescence. However, some of the features reported in literature were not in agreement with our studied old individuals such as the decrease in Naïve CD4^+^ T cells and CD4/CD8 ratio in addition to the increase in CD4^+^ and CD8^+^ central memory, CD8^+^ effector memory T cells, and pro-inflammatory cytokines (39, 47, 48). These differences may be due to the small number of individuals and great variability observed in the frequency of cell subtypes and cytokine production in our study population. Nonetheless, it cannot be ruled out by the possibility that the features preserved in immune system and observed in the old individuals studied are the key to achieve the longevity (13, 49, 50). Arai et al. (49) found in two different Japanese cohorts (*n* = 1,554) evaluating very old individuals (85–99 years), centenarians, and individuals ≥105 years that the lowest levels of inflammation correlated with the main indicators markers of successful aging, such as survival, capability, and cognition.

In old individuals, we observed lower rates of proliferation in CD4 and CD8 compartments in addition to the reduced levels of cytokines after stimulation with PHA. In association, we found lower percentage of CD4^+^ central memory T cells, which have been described as highly proliferative, and producers of IL-2 (13, 49–53). Corroborating with our data, Whisler et al. (51) showed that in *in vitro* there were diminished proliferative capacity and decreased production of IL-2 after stimulation with PHA in 7 out of 12 old individuals evaluated (mean age 78 years) in association with deficient activation of transcriptional factor AP-1 and nuclear factor of activated T cells.

Others have reported that age interferes negatively with the expansion of T cells due to telomere erosion (52, 53). On the other hand, after vaccination with live attenuated virus varicella zoster Qi et al. (54) observed that the majority of activated T cells were CD4^+^ and age (50–70 years) did not interfere with the expansion of antigen-specific T cells. However, the long-lived memory T cells (production of IFN-γ *in vitro*) decreased from day 14 to 28 post-vaccination.

The study of Shahid et al. (55) showed reduced expression of IFN-γ and granzyme B in CD8^+^ T cells of older adults vaccinated against influenza.

Our findings show that despite the common features of immune senescence presented by old individuals, they managed to achieve longevity.

Health professionals have proposed alternatives to circumvent age-associated diseases, such as physical activity, diet control, supplements, and probiotics (22–24). Vitamin D has been recommended due to its action on the immunity, but data from literature are inconclusive regarding the benefits of supplementation with vitamin D.

The US Endocrine Society defined vitamin D levels of 20 ng/mL or less as deficiency, 21–29 ng/mL as insufficiency, and 30 ng/mL or more as sufficiency (56). However, sub-optimal levels of vitamin D have been reported worldwide and depending on the lifestyle and environmental conditions, hypovitaminosis D could be observed in all age groups (57). In old adults, the diminished sun exposure, skin atrophy with decreased amounts of the precursor 7-dehydrocholesterol, and the reduced content of vitamin D in the diet leads to lower serum levels of vitamin D (58, 59). In accordance, blood samples were collected in summer and yet we observed that 50% of individuals older than 80 years showed vitamin D deficiency, while the insufficiency was observed in 50% of young individuals.

In innate immunity, macrophages and dendritic cells can convert vitamin D3 on biological active 1,25OH (60). In addition, immune cells also express the vitamin D receptor (VDR) and thus 1,25OH can act on immune microenvironment in paracrine and autocrine pathways (61).

Human monocytes activated through TLR upregulate the expression of genes associated with the conversion of 25OH to 1,25OH (Cyp27B1) and VDR. The addition of vitamin D to monocytes in culture leads to upregulation of VDR downstream genes, such as the antimicrobial cathelicidin (62–64). In opposition, it was observed that in adaptive immunity, vitamin D added in culture abolished the production of IFN-γ and IL-17 by CD4^+^ memory T cells co-cultured with activated dendritic cells (pneumococci products) (65). Accordingly, Rode et al. (66) found that human CD4^+^ T cells stimulated with CD3/CD28 beads in the presence of 25OH or 1,25OH present reduced production of IFN-γ.

In this study, there was a trend toward negative correlation between the absolute number of MDSC and vitamin D levels (*r* = −0563 *p* = 0.089) in young individuals. This is an important finding, since there was a higher percentage of MDSC in old individuals with predominance of the granulocytic phenotype (CD33^+^CD11b^+^CD15^+^) that has been associated with some types of cancer in humans. In addition, these cells have recently been associated with frailty and Alzheimer’s disease (11, 67).

Interventions to circumvent the MDSC increase have been proposed such as the use of vitamin D to induce the differentiation of non-mature suppressive myeloid cells into mature effector non-suppressive cells (68). In patients (49–71 years old) with head and neck cancer, vitamin D3 (20, 40, 60 μg/day) decreased the number of progenitor cells with suppressive phenotype, promoted the proliferation of T cells after *in vitro* stimulation, and increased the levels of effector cytokines (IL-12 and IFN-γ) (69).

Regarding to the CD4^+^ T cell compartment there was no correlation with the levels of vitamin D which is in agreement with literature data showing that for young adults, vitamin D levels (70), or supplement (71, 72) previously to vaccination did not cause enhanced humoral immunity that is dependent of CD4^+^ T cells help. However, Khoo et al. (73) showed that during winter the level of vitamin D decreases and is associated with lower percentage of Naive CD4^+^ T cells suggesting a role played by vitamin D in this cell compartment.

Our data points for correlation of vitamin D levels with some parameters of CD8^+^ T cells. In old individuals, vitamin D had a positive correlation with total CD8^+^ T cells. Considering that we observed a trend toward lower percentage of CD8^+^ T cells in old individuals (Figure 2), vitamin D could be beneficial in preventing the decrease of this cell subtype. In young individuals, vitamin D levels correlated positively with the frequency of CD8^+^ effector memory T cells.

Another important result was the negative correlation between vitamin D and CD8 EMRA T cells in old individuals suggesting that higher levels of vitamin D would be linked to less accumulation of cells that have been described as a marker of senescence (74, 75).

There was no correlation of vitamin D levels and proliferation of T cells (CD4^+^ and CD8^+^) or production of cytokines after stimulation with PHA. In agreement, the addition of vitamin D to culture of T cells stimulated with CD3 and CHO-CD80 cell line did not increase the proliferative capacity (individuals from 32 to 57 years old) (76). In addition, PBMCs stimulated *in vitro* in the presence of vitamin D showed diminished IFN-γ and increased IL-4 production in culture supernatant (77).

Aging has been related to chronic low-grade inflammation (inflammaging) with increased levels of circulating C-reactive protein (CPR), IL-6, and TNF-α. The InCHIANTI study found that individuals (*n* = 867, mean age 75.1 years) with vitamin D levels lower than 31.4 nmol/L presented high circulating IL-6, but not TNF-α, IL-1α, and IL-18 (78).

The English Longitudinal Study of Aging assessed community-dwelling individuals (*n* = 5,870, 50–80 years) and reported that low levels of 25OH (≤30 nmol/L) were negatively associated with CPR (79). A follow-up of old individuals (*n* = 23, 55–86 years) for 12 months showed that vitamin D levels were significantly lower in the winter with an increase in the number of individuals presenting deficiency. In the same season, there was a significant increase of circulating IL-6, IL-8, IL-β-1, MCP-1, and TNF-α (80). The low-grade chronic inflammation has been associated with aging-related diseases, and suboptimal levels of vitamin D have been related to chronic diseases/overall mortality (81–84), suggesting that adequate levels of vitamin D could benefit the aging population.

Despite the size of the sample be a limitation of the study and not allow more detailed statistical analyses, after testing the variables for normality and applying the adequate statistics, we obtained some important results. We found that the old population evaluated could be considered healthy based on the metabolic parameters. In this sample, 11 out of 12 were CMV^+^ and still maintained preserved some features of immunity such as CD4/CD8 ratio, and low production of inflammatory cytokines after stimulus. On the other hand, we observed increased frequency of MDSC, reduced number of circulating leukocytes, reduced percentage of total CD8^+^ and Naïve CD8^+^ T cells, and increased percentage of terminally differentiated CD8^+^EMRA T cells. CMV^+^ was correlated with the decrease of CD8^+^ Naïve T cells and increase in CD8^+^ EMRA T cells. Vitamin D levels were insufficient in 50% of old individuals and correlated positively with total CD8^+^ T cells and negatively with CD8 EMRA T cells. Our next step is to develop an *ex vivo* model to study the action of vitamin D in CD4^+^ and CD8^+^ T cells, associated phenotypes, proliferation, and cytokines production.

## Conclusion

In the studied population, longevity was correlated to maintenance of some immune parameters. Considering the limitations of the study as size of the sample and lack of functional assays showing the direct effect of vitamin D in immunity, it was found that vitamin D in old individuals was correlated to some features of the immune system, mainly in the CD8 compartment.

## Ethics Statement

This study was carried out in accordance with the recommendations of “UNIFESP CEP Committee of Ethics in Research” with written informed consent from all subjects. All subjects gave written informed consent in accordance with the Declaration of Helsinki. The protocol was approved by the “CEP Committee of Ethics in Research.”

## Author Contributions

All authors contributed significantly to this study and have read and approved the submitted manuscript. AA—laboratorial experiments, data analysis, figures. MI—laboratorial experiments. YD—SABE coordinators of study teams responsible for recruitment and sample collection. VB—laboratorial experiments and data analysis, figures, manuscript writing, and primary responsibility for final content.

## Conflict of Interest Statement

The authors declare that the research was conducted in the absence of any commercial or financial relationships that could be construed as a potential conflict of interest.
